# Early-time-point ^18^F-FDG-PET/CT and other prognostic biomarkers of survival in metastatic melanoma patients receiving immunotherapy

**DOI:** 10.2478/raon-2025-0014

**Published:** 2025-02-27

**Authors:** Nezka Hribernik, Katja Strasek, Andrej Studen, Katarina Zevnik, Katja Skalic, Robert Jeraj, Martina Rebersek

**Affiliations:** 1 Department of Medical Oncology, Institute of Oncology Ljubljana, Ljubljana, Slovenia; 2 Faculty of Medicine, University of Ljubljana, Ljubljana, Slovenia; 3 Faculty of Mathematics and Physics, University of Ljubljana, Ljubljana, Slovenia; 4 University of Wisconsin Carbone Cancer Centre, Madison, WI, USA; 5 Department of Medical Physics, University of Wisconsin-Madison, Madison, WI, USA; 6 Experimental Particle Physics Department, Jozef Stefan Institute, Ljubljana, Slovenia; 7 Department of Nuclear Medicine, Institute of Oncology Ljubljana, Ljubljana, Slovenia

**Keywords:** early time-point ^18^F-FDG-PET/CT, prognostic biomarkers, immune-related adverse effects metastatic melanoma, immune-checkpoint inhibitor

## Abstract

**Background:**

A considerable proportion of metastatic melanoma (mM) patients do not respond to immune checkpoint inhibitors (ICIs). There is a great need to develop noninvasive biomarkers to detect patients, who do not respond to ICIs early during the course of treatment. The aim of this study was to evaluate the role of early [^18^F]2fluoro-2-deoxy-D-glucose PET/CT (^18^F-FDG PET/CT) at week four (W4) and other possible prognostic biomarkers of survival in mM patients receiving ICIs.

**Patients and methods:**

. In this prospective noninterventional clinical study, mM patients receiving ICIs regularly underwent ^18^F-FDG PET/CT: at baseline, at W4 after ICI initiation, at week sixteen and every 16 weeks thereafter. The tumor response to ICIs at W4 was assessed via modified European Organisation for Research and Treatment of Cancer (EORTC) criteria. Patients with progressive metabolic disease (PMD) were classified into the no clinical benefit group (no-CB), and those with other response types were classified into the clinical benefit group (CB). The primary end point was survival analysis on the basis of the W4 ^18^F-FDG PET/CT response. The secondary endpoints were survival analysis on the basis of LDH, the number of metastatic localizations, and immune-related adverse events (irAEs). Kaplan-Meier analysis and univariate Cox regression analysis were used to assess the impact on survival.

**Results:**

Overall, 71 patients were included. The median follow-up was 37.1 months (952% CI = 30.1–38.0). Three (4%) patients had only baseline scans due to rapid disease progression and death prior to W4 ^18^F-FDG-PET/CT. Fifty-one (72%) patients were classified into the CB group, and 17 (24%) were classified into the no-CB group. There was a statistically significant difference in median overall survival (OS) between the CB group (median OS not reached [NR]; 95% CI = 17.8 months – NR) and the no-CB group (median OS 6.2 months; 95% CI = 4.6 months – NR; p = 0.003). Univariate Cox analysis showed HR of 0.4 (95% CI = 0.18 – 0.72; p = 0.004). median OS was also significantly longer in the group with normal serum LDH levels and the group with irAEs and cutaneous irAEs.

**Conclusions:**

Evaluation of mM patients with early ^18^F-FDG-PET/CT at W4, who were treated with ICIs, could serve as prognostic imaging biomarkers. Other recognized prognostic biomarkers were the serum LDH level and occurrence of cutaneous irAEs.

## Introduction

Immunotherapy with immune checkpoint inhibitors (ICIs) has greatly impacted the treatment landscape of metastatic melanoma (mM) patients. Final, 10-year results from pivotal randomized clinical trial have shown ongoing benefits, with 10-year overall survival (OS) rates of 34%, 37%, and 43% in mM patients receiving pembrolizumab, nivolumab, and ipilimumab/nivolumab, respectively.^1,2^ However, a considerable proportion of mM patients do not respond to ICIs.

Normal serum levels of lactate dehydrogenase (LDH) and a small number of organs with metastatic involvement are two very strong and well-recognized prognostic biomarkers of better survival in mM patients.^3–5^ In addition, immune-related adverse events (irAEs) have been proven in some studies to be biomarkers of improved response rates and longer survival in patients treated with ICIs.^6,7^ Specifically, endocrine and cutaneous irAEs were associated with favourable survival outcomes compared with patients without this type of irAE in a large retrospective multicohort study.^6^ There is a great need to develop other noninvasive imaging biomarkers (IBM) to detect mM patients who do not respond to ICIs early in the course of treatment.

Positron emission tomography/computed to-mography with [^18^F]2fluoro-2-deoxy-D-glucose PET/CT (^18^F-FDG PET/CT) is a noninvasive method that combines anatomical and functional data. It is generally used as a diagnostic tool for the staging of melanoma patients and is now gaining valuable value in immunotherapy treatment response evaluation and prognosticating outcomes.^7,8^ In addition to staging and response monitoring, ^18^F-FDG PET/CT has also shown some potential for detecting immune-related side effects (irAEs), such as the use of organ ^18^F-FDG uptake, quantified by percentiles of standardized uptake values (SUV) distribution as a quantitative IBM of irAEs.^9–11^

The optimal timing of the first on-treatment ^18^F-FDG PET/CT evaluation of mM patients on ICIs is a matter of ongoing investigations. Joint EANM/SNMMI/ANZSNM practice guidelines/procedure standards recommended an interim ^18^F-FDG PET/CT scan during immunomodulatory treatment in patients with solid tumors at 8–12 weeks after the start of treatment (i.e., after 3–4 cycles of immunotherapy).^7^ However, there are studies analysing ^18^F-FDG PET/CT scans performed earlier after ICI initiation. In a prospective study with 20 mM patients treated with ipilimumab, ^18^F-FDG PET/CT was performed between 21-28 days after treatment start. They concluded that the combination of changes in lesion dimensions along with changes in ^18^F-FDG uptake may be associated with immune activation and a favourable outcome.^12^

We previously reported the results of our prospective study regarding the role of quantitative IBM in early ^18^F-FDG PET/CT, which was performed four weeks after ICI initiation, for the detection of immune-related adverse events in melanoma patients.^10^ Here, we evaluated the role of early ^18^F-FDG-PET/CT at week four and other possible prognostic biomarkers of survival in mM patients receiving ICIs.

## Patients and methods

### Patients

We enrolled patients 18 years of age or older who had histologically confirmed, unresectable, advanced melanoma and were planned to be treated per standard of care with ICIs with anti-cytotoxic T-lymphocyte-associated antigen 4 (anti-CTLA-4) and/or anti-programmed death-1 (anti-PD-1) treatment in the first or second line of systemic treatment at the Institute of Oncology Ljubljana, Slovenia. The key exclusion criteria included symptomatic brain metastases and malignant diseases other than melanoma.

### Trial design

In this noninterventional, prospective study, patients underwent baseline ^18^F-FDG PET/CT within four weeks before treatment initiation and were monitored regularly with serial ^18^F-FDG PET/CT: at week four (W4) (+/- 5 days), week 16 (W16) (+/- 7 days), and week 32 (W32) (+/- 7 days) after treatment initiation and every 16 weeks thereafter. The first follow-up ^18^F-FDG PET/CT at W4 was performed for investigational purposes and was not necessarily used to guide treatment decisions. The clinical data and images included in this analysis were obtained from disease diagnosis up to 1^st^ September 2024.

All ^18^F-FDG PET/CT data were acquired before and during ICI treatment, and all clinical data were collected for review. The irAE grade was assigned prospectively and scored with the use of the National Cancer Institute Common Terminology Criteria for Adverse Events (CTCAE, v.5.0).^13^ The irAE were classified as serous irAE in case of higher grade of irAE of 3 and above. Imaging and clinical data were anonymized and stored in a secure LabKey database server.^14^

The clinical protocol was approved by the Ethics Committee ERIDEK-0034/2020 and the Clinical Trials Protocol Review Committee ERID-KSOPKR-0032/2020 at the Institute of Oncology Ljubljana and by the Commission of the Republic of Slovenia for Medical Ethics (approval number: 0120-256/2020-14, September 15^th^ 2020). It was conducted following the ethical standards defined by the Declaration of Helsinki and the International Conference on Harmonization Guidelines for Good Clinical Practice. The study was registered with ClinicalTrials.gov under the registration number NCT06207747.

The study was conducted with the acknowledgment and consent of the subjects. All patients provided signed informed consent for treatment and consent allowing the use of their data for scientific purposes.

### ^18^F-FDG PET/CT acquisition and analysis

All ^18^F-FDG PET/CT scans were obtained on Biograph mCT PET/CT (Siemens, Knoxville, TN). Imaging protocol required patients to fast for 6 hours prior to injection of the radiotracer and have a blood glucose level below 10 mmol/L at the time of the scan. Patients were required to hold all diabetic medication, including metformin, for 6 hours prior to radiotracer injection. All scans were acquired per standard of care. CT that meets response evaluation criteria in solid tumours (RECIST) analysis needs was acquired according to adjusted protocol including sinogram affirmed iterative reconstruction (SAFIR) to minimize dose. Following reconstruction, PET images were normalized by patient weight and injected dose to compute SUV. More details about image acquisition can be found in our previous paper, where the same cohort of patients was used for analysis.^11^

The tumor response to ICIs on ^18^F-FDG PET/CT was evaluated by a nuclear medicine specialist combining the European Organisation for Research and Treatment of Cancer (EORTC) criteria and visual response assessment.^15^ The SUV_max_ and size of the lesions were measured in all most representative tumor lesions, which are the largest lesions of a certain area or organ with the highest FDG uptake at baseline, W4 and all consecutive ^18^F-FDG PET/CT scans. Patients were classified into four major categories on the basis of the tumor response to ICIs: complete metabolic response (CMR), partial metabolic response (PMR), stable metabolic disease (SMD) and progressive metabolic disease (PMD).^15^ According to the information gathered after the whole -body ^18^F-FDG PET/CT visual assessment, two new categories, of heterogenous response (HGR) and possible pseudo-progressive disease (PPD), were added for tumor response evaluation. HGR was assigned when multiple lesions were variably meeting the criteria of PMD, SMD, PMR and CMR and could not be classified into only one response evaluation category. PPD was assigned in the case of moderate metabolic progression of the baseline tumor lesions with few locally distributed new lesions. Obvious progression with multiple new tumor lesion sites was classified as true progression (PMD). The patients were further stratified into two groups: patients with PMD were classified into the no clinical benefit (no-CB) group and patients with other response categories into the clinical benefit (CB) group. A summary of the response criteria is presented in Supplementary Table 1.

**TABLE 1.** Patient demographics, cancer staging, treatment details, and outcomes

Characteristics	No = 71 (%)
Age; mean (+/-SD) (yr)	62 ± 12
Gender	
Male	43 (61)
Female	28 (39)
ECOG performance status	
0	30 (42)
1	41 (58)
AJCC	
III.D	1 (1)
M1a	16 (23)
M1b	10 (14)
M1c	32 (45)
M1d	12 (17)
Anatomic site of primary	
Cutaneous	58 (82)
Ocular	4 (6)
Mucosal	3 (4)
Unknown primary	6 (8)
Line of systemic treatment for metastatic disease	
1^st^ line	63 (89)
2^nd^ line	8 (11)
Baseline LDH	
Elevated	23 (32)
Normal	49 (68)
Number of organs with metastatic involvement	
1	25 (35)
2	21 (30)
3	11 (15)
> 3	14 20)
Actionable mutation	
*BRAF* wild type	21 (30)
*BRAF* V600E	28 (39)
*BRAF* V600K	10 (14)
*BRAF* V600 - others	1 (1)
*NRAS*	11 (16)
Type of systemic treatment	
PD-1 inhibitors	47 (66)
Combination of PD-1 and CTLA-4 inhibitors	24 (34)
Tumor response on week 4 ^18^F-FDG PET/CT	
Complete metabolic response	3 (4)
Partial metabolic response	12 (17)
Stable metabolic disease	10 (14)
Heterogenous response	6 (8)
Possible pseudoprogression	20 (28)
Progressive metabolic disease	17 (24)

1 AJCC = American Joint Classification of Cancer; BRAF = V-Raf murine sarcoma viral oncogene homolog B; CTLA-4 = Cytotoxic T lymphocyte-associated antigen 4; ECOG = Eastern Cooperative Oncology Group; ICI = Immune checkpoint inhibitors; No = number of patients; NRAS = neuroblastoma RAS viral homolog; PD-1 = programmed death-1; SD = standard deviation

### Outcomes and statistical analysis

The primary end point of this study was the analysis of median OS based on the W4 ^18^F-FDG PET/CT response. The secondary endpoint was the median OS, which was analysed on the basis of the level of LDH, the number of organs with metastatic involvement at the beginning of ICI treatment, occurrence of irAE, higher irAE, cutaneous irAEs, endocrine irAEs and immune-related thyroiditis (irThyroiditis).

Patient characteristics were summarized via descriptive statistics. Survival analysis was performed via the Kaplan–Meier method, and 95% confidence intervals (CIs) were calculated. The associations of each of the eight metrics with OS were assessed with a univariate Cox proportional hazard model. With the use of the Bonferroni correction for testing multiple hypothesis, probability values p < 0.006 were considered statistically significant.

## Results

From September 2020 through September 2022, a total of 71 patients were enrolled. The characteristics of the patients are summarized in Table 1. At the cut-off date of the observational period for this analysis on 1^st^ September 2024, the median follow-up was 37.1 months (95% CI = 30.1-38.0). The median duration of ICI therapy was 10.2 months (range: 1-39.4 months). Three (4%) patients had only baseline scans due to rapid disease progression and death prior to W4 ^18^F-FDG-PET/CT. The timing of ^18^F-FDG PET/CT relative to ICI treatment initiation and the number of ^18^F-FDG-PET/CT images are shown in the Supplementary Table 2.

**TABLE 2.** Clinical diagnosis of immune-related adverse events

Immune-related adverse event	Any grade No (%)	Grade 3-5 No (%)	Time to onset of irAE (mean ± SD) [weeks]
No. of pts with at least one irAE	56 (79)	13 (18)	-
Number of all irAE events	144	14	144 ± 161
Gastrointestinal			
Diarrhea	14 (20)	2 (3)	31.7 ± 30.6
Colitis	7 (10)	2 (3)	39 ± 32
Xerostomia	2 (3)	0	30.1 ± 4.4
Gastritis	2 (3)	0	64.4 ± 7.7
Stomatitis	1 (1)	0	3 ± 0
Respiratory			
Pneumonitis	5 (7)	0 (0)	40.9 ± 45.7
Sarcoid reaction	2 (3)	0 (0)	6.3 ± 0.3
Hepatic			
Increased AST/ALT	16 (23)	4 (6)	7.7 ± 8.3
Endocrine			
Hypothyroidism	10 (14)	0	12.3 ± 6
Hyperthyroidism	7 (10)	0	7.3 ± 7.1
Adrenal insufficiency	2 (3)	2 (3)	34.4 ± 20.1
Diabetes mellitus	1 (1)	1 (1)	72.7 ± 0
Pancreatitis	1 (1)	1 (1)	32.4 ± 0
Cutaneous			
Pruritus	23 (32)	0	10.7 ± 10
Skin rash	16 (23)	0	14 ± 16.3
Vitiligo	9 (13)	0	37.4 ± 31.6
Poliosis of hair	1 (1)	0	34.7 ± 0
Musculoskeletal			
Arthritis	10 (14)	0	28.7 ± 27.3
Myalgia	2 (3)	0	16.7 ± 12.3
Arthralgia	1 (1)	0	14 ± 0
Synovitis	1 (1)	0	65.4 ± 0
Neurological			
Encephalitis	2 (3)	1 (1)	36.4 ± 32.9
Psychosis	1 (1)	0	25.4 ± 0
Other			
Fatigue	6 (8)	0	6.5 ± 4.9
Hypophosphatemia	1 (1)	0	17 ± 0

1 AST/ALT = aspartate transaminase/alanín aminotransferaza; irAE = immune-related adverse events

### Survival outcomes

**FIGURE 1.**
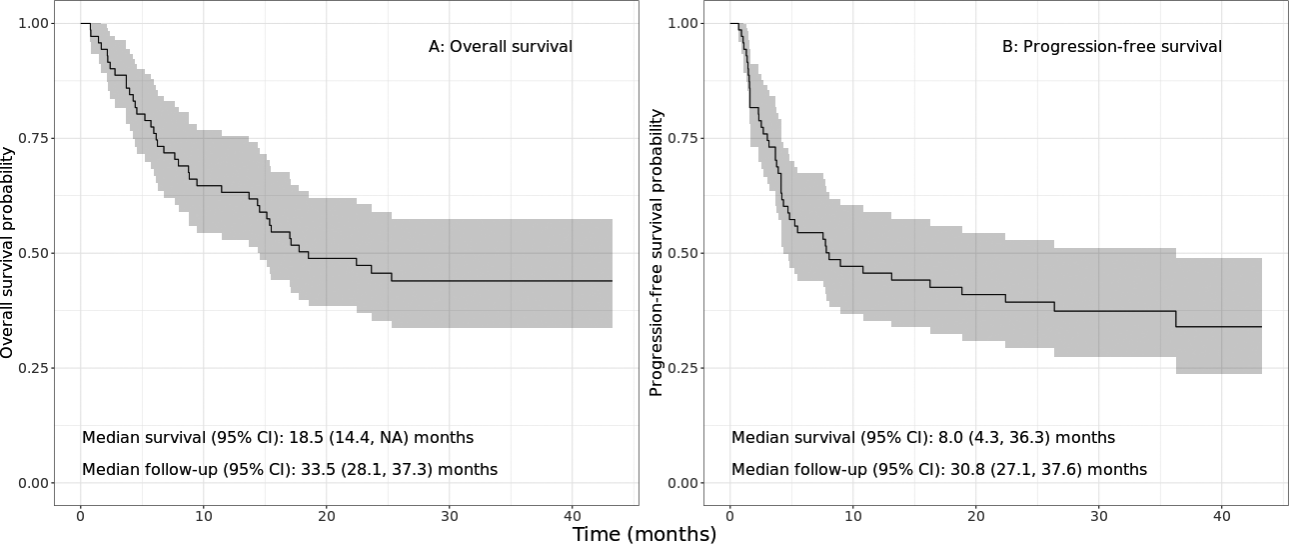
Kaplan-Meier curves show overall survival (OS) probability **(A)** and progression-free survival (PFS) probability **(B)** over time in patients with metastatic melanoma treated with immune checkpoint inhibitors. The grey shading reflects the 95% confidence interval.

**FIGURE 2.**
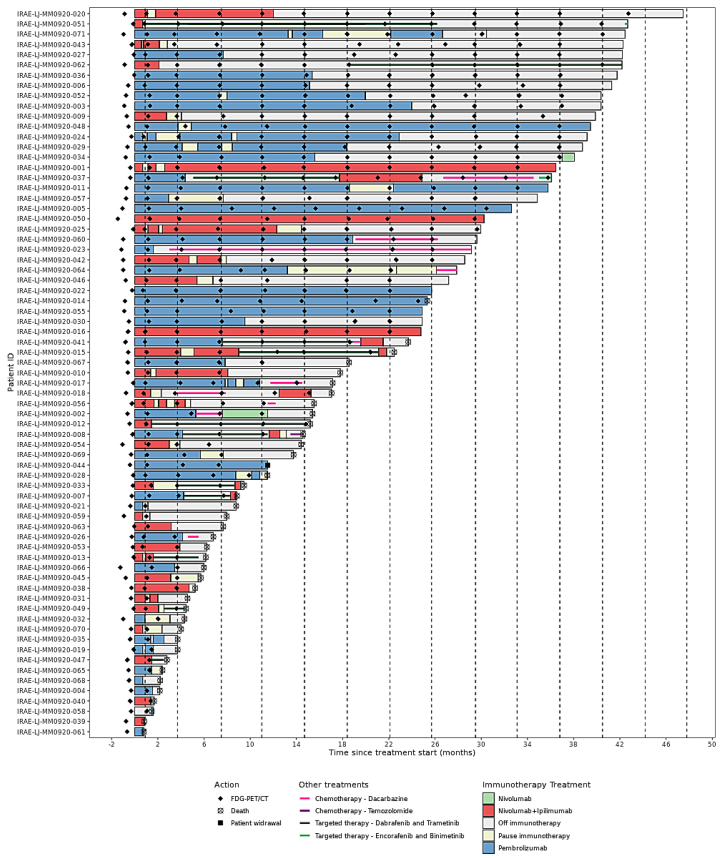
Swimmer plot shows individual patient’s treatment progression in each horizontal line. Colourful bars and lines indicate type and duration of treatment, while dots indicate specific action - ^18^F-FDG-PET/CT imaging or reason for end-of-study (if applicable). Vertical dashed lines indicate a time when ^18^F-FDG-PET/CT scan should be performed for patients in this study.

The Kaplan–Meier estimated OS and progressionfree survival (PFS) for the whole patient group were 18.5 months (95% CI = 14.4 months – not reached [NR]) and 8.1 months (95% CI = 4.3–26.3 months), respectively (Figure 1). Among the whole group, 39 (55%) patients died, and 44 (62%) patients progressed to immunotherapy before the cut-off date. On Figure 2, the swimmer plot displays data for individual patients, where each horizontal line or bar shows type and duration of treatment, and each point represents either ^18^F-FDG-PET/CT or end-of-study reason (if applicable).

### W4 ^18^F-FDG PET/CT and survival outcomes

**FIGURE 3.**
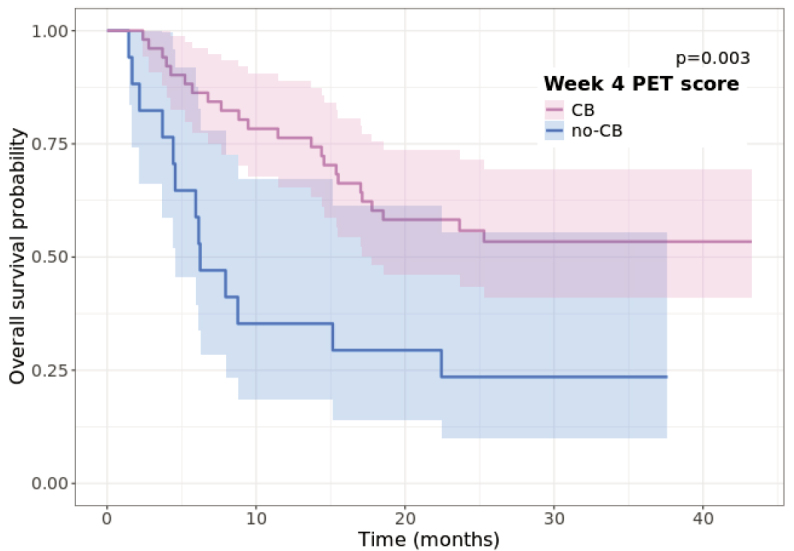
Kaplan-Meier curves showing probability of median overall survival (OS) between clinical benefit (CB) and no-CB group as classified by findings on week four (W4) ^18^F-FDG PET/CT. The curves are statistically significantly different (p = 0.03).

Among the 68 (96%) patients who underwent W4 ^18^F-FDG PET/CT, 51 (72%) patients were classified into the CB group, and 17 (24%) were classified into the no-CB group. The median OS was not reached (NR) (95% CI = 17.8 months - NR) in the CB group and was 6.2 months (95% CI = 4.6 months - NR) in the no-CB group (Figure 3). In univariate Cox analysis classification was statistically significantly correlated to OS with hazard ratio (HR) of 0.4 (95% CI = 0.18–0.72; p = 0.004).

Among the 17 patients with PMD, who were classified into the no-CB group, 7 (42%) died before the W16 ^18^F-FDG PET/CT scan. Three (18%) patients had PMD on W16 ^18^F-FDG PET/CT, 6 (35%) had PMR, and one (6%) patient had CMR. Two (12%) patients with PMD on W4 ^18^F-FDG PET/CT changed systemic therapy from ICI therapy to targeted therapy because of clinical and radiological signs of rapid progression affecting vital organs.

Three patients were classified as CMR on W4 ^18^F-FDG PET/CT. One patient achieved a durable response with CMR, one patient experienced fatal grade 5 immune-related encephalitis during treatment and one experienced local progression in soft tissues 25 months after CMR imaging on W4 ^18^F-FDG PET/CT. The site of progression was amenable for local treatment with radiotherapy, and a complete metabolic response was achieved.

**FIGURE 4.**
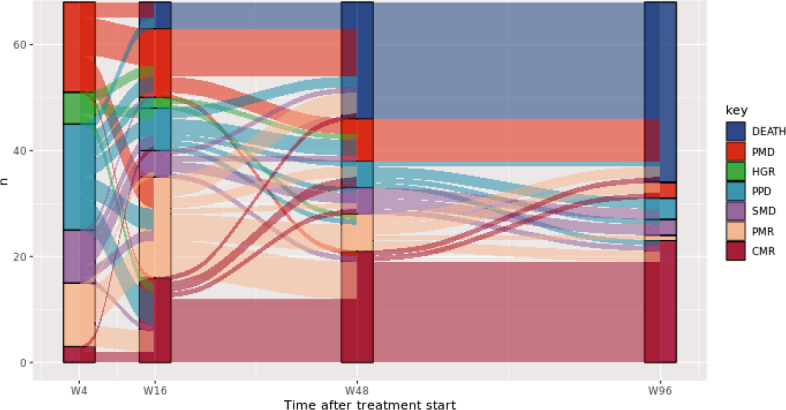
Alluvial plot illustrates the flow of patients between different response categories on ^18^F-FDG PET/CT scan across four evaluation time points: at week 4 (W4), week 16 (W16), week 48 (W48) and week 96 (W96). CMR = complete metabolic response; HGR = heterogeneous response; PMD = progressive metabolic disease; PPD = pseudoprogressive disease; SMD = stable metabolic disease; PMR = partial metabolic response; n = number of patients

In Figure 4, the alluvial plot shows the responses on ^18^F-FDG PET/CT scans for each patient at week 4, 16, 48 and 96.

### Other biomarkers and survival outcomes

#### LDH and the number of organs with metastatic involvement

**FIGURE 5.**
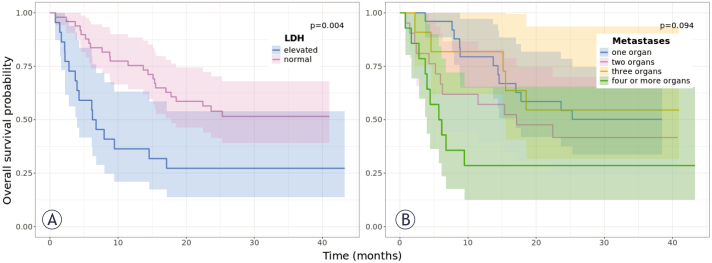
Kaplan-Meier curves show the median overall survival (OS) probability of patients with metastatic melanoma treated with immune checkpoint inhibitors according to **(A)** LDH level and **(B)** the number of organs with metastatic involvement. The shading reflects the 95% confidence interval.

Twenty-three (32%) patients had elevated serum LDH at ICI initiation. The median OS of patients with normal LDH levels was NR (95% CI = 17.8 months - NR), and that of patients with elevated LDH levels was 6.5 months (95% CI = 4.0 months - NR). The difference in OS was statistically significant between these two groups (p = 0.004) (Figure 5A); Univariate Cox analysis showed a HR = 0.4 (95% CI = 0.21–0.76; p = 0.005).

The difference in OS based on the number of organs with metastatic involvement was not statistically significant (p = 0.094) (Figure 5B).

### Immune-related adverse events (irAEs)

Among the 71 included patients, 56 (79%) developed irAEs, including 13 (18%) with grade 3 or higher irAEs. All irAE, their number and time to onset, are presented in Table 2. One (2%) patient died of immune-related encephalitis. Due to irAEs, 7 (10%) patients were hospitalized. Three (4%) patients were diagnosed with autoimmune disease prior to ICI initiation: one had vitiligo, one had scalp psoriasis, and one was diagnosed with rheumatoid arthritis at the time of ICI initiation. None of them experienced an exacerbation of their autoimmune disease or needed special treatment for that reason.

**FIGURE 6.**
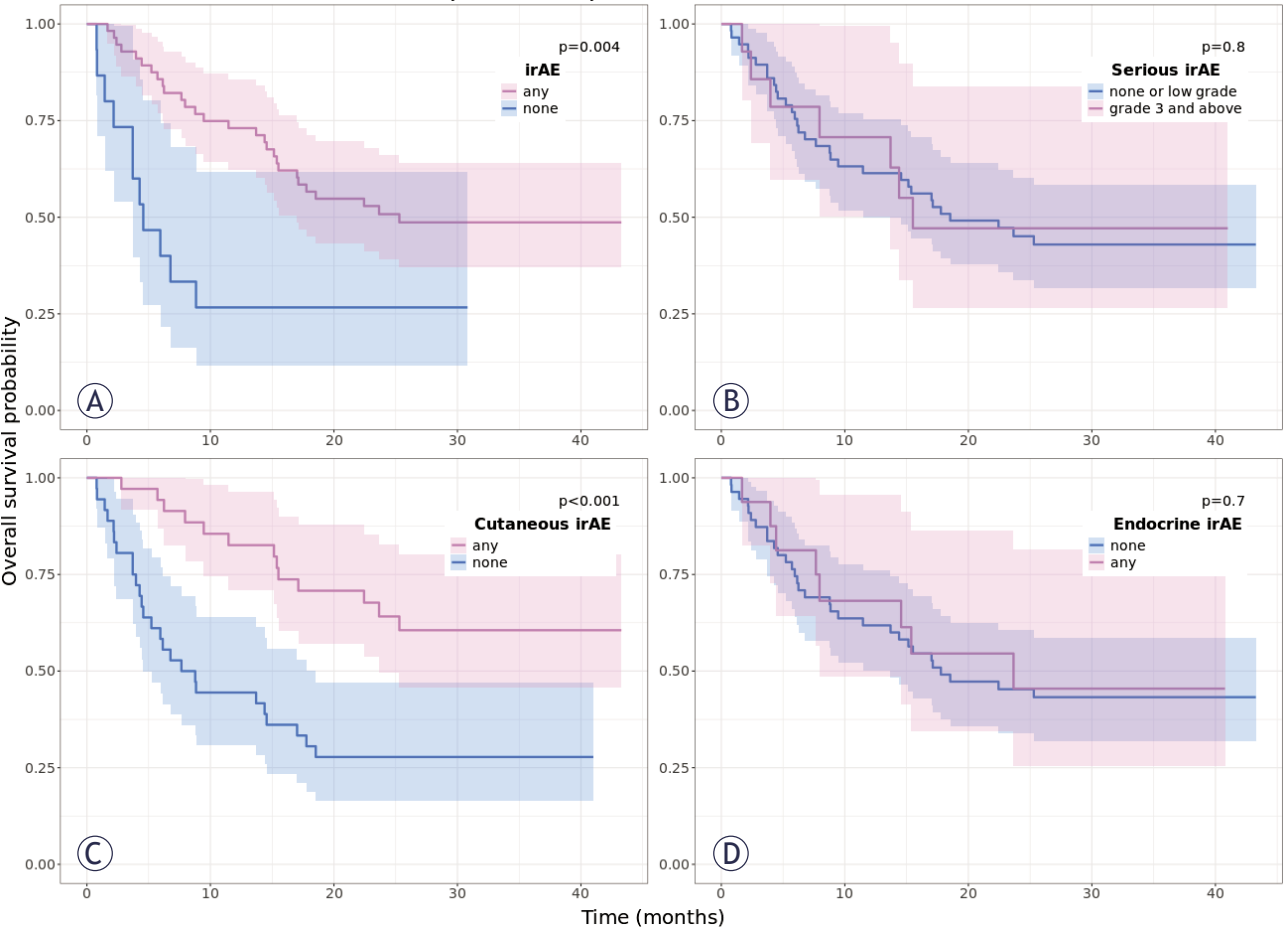
Kaplan-Meyer curves of the median overall survival **(OS)** over time in patients with metastatic melanoma treated with immune checkpoint inhibitors according to **(A)** occurrence of immune-related adverse events, **(B)** occurrence of serious immune-related adverse events, **(C)** cutaneous immune-related side effects, **(D)** immune-related endocrine immune-related side effects. The blue and pink shading reflects the 95% confidence intervals for respecting groups.

The median OS was 25.3 months (95% CI = 17 months - NR) in patients with irAEs and 4.6 months (95% CI = 3.7 months - NR) in patients without irAEs (p = 0.004) (Figure 6A). Univariate Cox analysis showed a HR 0.9 (95% CI = 0.18–0.75; p = 0.006). The median OS was not reached (95% CI = 23.7 months - NR) in patients who experienced cutaneous irAEs and was 8.2 months (95% CI = 4.6–17.8) in patients without cutaneous irAEs (p < 0.0001) (Figure 6C); Cox analysis showed a HR 0.36 (95% CI = 0.19–0.66; p = 0.001). Using Kaplan-Meier analysis, a significant statistical difference in OS was not observed between patients with and without higher-grade irAEs (p = 0.783) (Figure 6B), endocrine irAEs (p = 0.7) (Figure 6D) or immunerelated thyroiditis (p = 0.711) (Supplementary Figure 1).

**FIGURE 7.**
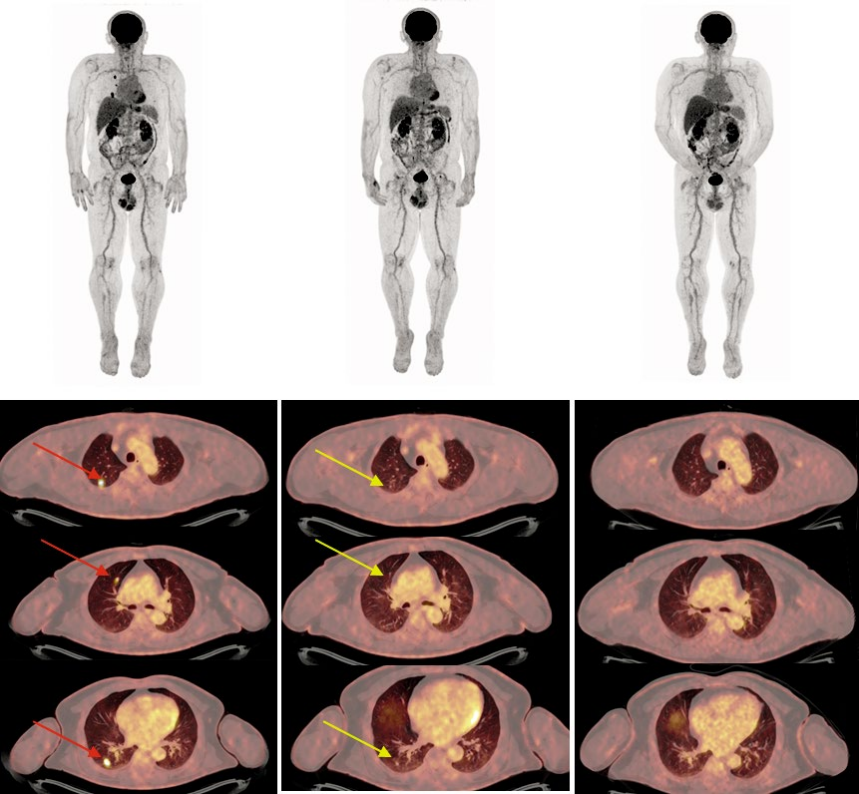
A 67-year-old male patient, diagnosed with metastatic NRAS-mutated cutaneous melanoma with lung metastases in January 2021, was treated with pembrolizumab in the first-line setting. Serial 18F-FDG PET/CT scans were obtained per the study protocol. The images above show the maximal intensity projection (MIP) on the baseline PET/CT (left), on the week 4 evaluation PET/CT (middle) and at the endpoint of the study (right). The images below show transverse sections of the lungs in different planes, revealing three FDG-avid metastatic nodules in the right lung (lower left images, red arrows), only small nodules with no FDG uptake on week 4 PET/CT (lower middle images, yellow arrows), a complete metabolic response, and no residual nodules found at the end point of the PET/CT images with persistent complete remission (lower right images).

## Discussion

The evaluation of mM pts with early ^18^F-FDG-PET / CT at W4, when treated with ICIs, can serve as a survival imaging biomarker (IBM). Based on our results, patients with no-CB at W4 had a shorter survival compared with the CB group (p = 0.001). This was also observed in the study by Cho *et al*., where early ^18^F-FDG-PET/CT scans of 20 patients 21–28 days after treatment started showed a predictive role for response. Unlike in our group, patients in their study were mostly treated with the CTLA-4 inhibitor ipilimumab and tumor response was assessed according to RECIST, immune-related response criteria, PERCIST and EORTC criteria.^12^ In another study by Anderson *et al*., ^18^F-FDG-PET/CT was performed after a single dose of pembrolizumab, at a median of 7 days (range: 3–21 days) after the start of treatment. They concluded that early scan could identify metabolic changes in metastases that are potentially predictive of response to ICIs.^16^ Additionally, in recent studies on neoad-juvant immunotherapy in melanoma patients, responses were reported as early as two weeks after ICI initiation, and pathological responses were reported at 4-6 weeks after treatment start.^17,18^ The use of this strategy with early evaluation is not yet fully understood, but it may lead to new imaging evaluation strategies for patients undergoing immunotherapy.

Seven (10%) patients in our study who were classified as PMD on W4 ^18^F-FDG-PET/CT showed a later response, as seen on subsequent ^18^F-FDG PET/CT scans (Figure 4). For this subgroup of patients, early cessation of ICI therapy has a detrimental effect. More analysis is needed to identify optimal early timepoint scans and to find more specific biomarkers, perhaps using artificial intelligence with automated deep learning-based lesion segmentation, to distinguish patients and lesions that are in progress from those patients who are just late responders to ICIs.^19,20^

^18^F-FDG-PET/CT seems to play an important role not only at the early beginning of treatment with ICIs at W4 but also later during treatment. In a retrospective analysis of 104 patients with baseline and 1-year CT and PET/CT scans, Dimitriou *et al*. reported that almost all patients with CMR at one year had an ongoing response to ICIs thereafter.^8^ In our cohort of patients, most of the patients with CMR at week 42 remained in remission, as shown in the alluvial plot in Figure 4. The case presentation in Figure 5 shows a patient with early and long-lasting CMR.

In our cohort, occurrence of cutaneous irAEs was clearly associated with longer survival. Cutaneous irAEs, especially vitiligo, are more common in patients with melanoma than in other cancers. The higher frequency can be explained by shared immunogenic antigens between healthy tissue and tumors.^21,22^ Cutaneous irAEs are usually lower grade and vigorous immunosuppressive management is not necessary; therefore, there is no unfavorable effect of irAE management on ICI efficacy or survival.^22^ Contrary to the results from a large retrospective multicohort study^6^, our study proved no survival benefit for patients with irThyroiditis or endocrine irAEs, possibly due to the low number of patients with this type of adverse event. Further studies will clarify the prognostic role of this type of irAE.

Our study is limited by not performing analysis of circulating tumor DNA in plasma, not available at Institute of Oncology Ljubljana when the study started, and not using other volumetric PET parameters, like metabolic tumor volume or total lesion glycolysis.^16,23,24^ Another limitation of this study is that we did not perform lesion-level response analysis, which would provide even better insights into lesion-level and patient-level response patterns. Regarding response criteria to ICIs, a wide range of criteria have been proposed and compared in recent years: PERCIST, PECRIT, PERCIMT iPERCIST and imPERCIST5.^7,24–26^ As further evaluation of these newly proposed criteria is still warranted, our decision was to use standard EORTC criteria, adapted based on recommendations from the EANM/SNMMI/ANZSNM.

Whole-body PET imaging has great potential for future work, especially the use of artificial intelligence.^27^ In line with this, our future work will include segmentation of all disease with lesion-by-lesion analysis on W4 and later ^18^F-FDG-PET/CT images in our cohort of patients. With more indepth analysis, we hope to identify specific lesions that do not respond to treatment early in the start of the treatment and offer our patients more personalized treatment. Larger, possibly multicenter studies using same steps in analysis are needed to develop new biomarkers, including imaging biomarkers, to guide patient and treatment selection.^28^

## Conclusions

The evaluation of mM patients with early 18F-FDG-PET/CT at W4 who were treated with ICIs revealed a strong prognostic IBM. To obtain more information from early ^18^F-FDG-PET/CT, artificial intelligence will likely play an important role.

## Supplementary Material

Supplementary Material Details
