# Supplementary material for: Early-time-point 18F-FDG-PET/CT and other prognostic biomarkers of survival in metastatic melanoma patients receiving immunotherapy
Source: Radiol Oncol. 2025 Feb 27;59(1):43–53. doi: 10.2478/raon-2025-0014 (PMC11867565; doi:10.2478/raon-2025-0014)
Supplement: Supplementary file 1 — Supplementary Material Details [file raon-2025-0014_sm.pdf]

Early-time-point ¹⁸F-FDG-PET/CT and other prognostic biomarkers of survival in metastatic melanoma patients receiving immunotherapy

Nezka Hribernik, Katja Strasek, Andrej Studen, Katarina Zevnik, Katja Skalic, Robert Jeraj, Martina Rebersek

doi: 10.2478/raon-2025-0014

SUPPLEMENTARY TABLE 1. Response criteria

	Tumor response	Description
CB	Complete metabolic response (CMR)	Complete resolution of ^{18}F-FDG uptake within all the tumor lesions, so that the uptake of ^{18}F-FDG was indistinguishable from the surrounding normal tissue, or complete disappearance of the lesion
	Partial metabolic response (PMR)	Reduction of a minimum of 15-25% in tumor SUV_{max}
	Stable metabolic disease (SMD)	Increase in tumor SUV_{max} of less than 25% or a decrease of less than 15% and no visible increase in extent of ^{18}F-FDG tumour uptake (20% in the longest dimension)
	Mixed or heterogenous response (HGR)	Multiple lesions variably meeting the criteria of PMD, SMD, PMR and CMR and could not be classified in solely one response evaluation category
	Possible pseudoprogressive disease (PPD)	Moderate metabolic progression of the baseline tumor lesions with few locally distributed new lesions. Unconfirmed PMD that needs to be confirmed with another consecutive ^{18}F-FDG PET/CT scan.
No-CB	Progressive metabolic disease (PMD)	Increase in ^{18}F-FDG tumour SUV of greater than 25% within the tumor lesions defined on the baseline scan, visible increase in the extent of ^{18}F-FDG tumour uptake (20% in the longest dimension) or the appearance of new ^{18}F-FDG uptake in metastatic lesions

CB = clinical benefit

SUPPLEMENTARY TABLE 2. Timing of ^{18}F-FDG PET/CT relative to immune checkpoint inhibitors (ICI) treatment initiation

	Number of patients	Range [min-max] (days)	Mean (days)	Median (days)	SD (days)
Pre-Treatment	71	[-44, -1]	-16	-16	10
W4 (28 days)	68	[21, 62]	34	34	7
W16 (112 days)	55	[105, 150]	115	112	8
W32 (224 days)	48	[196, 280]	226	224	12

SD = standard deviation; W = Week

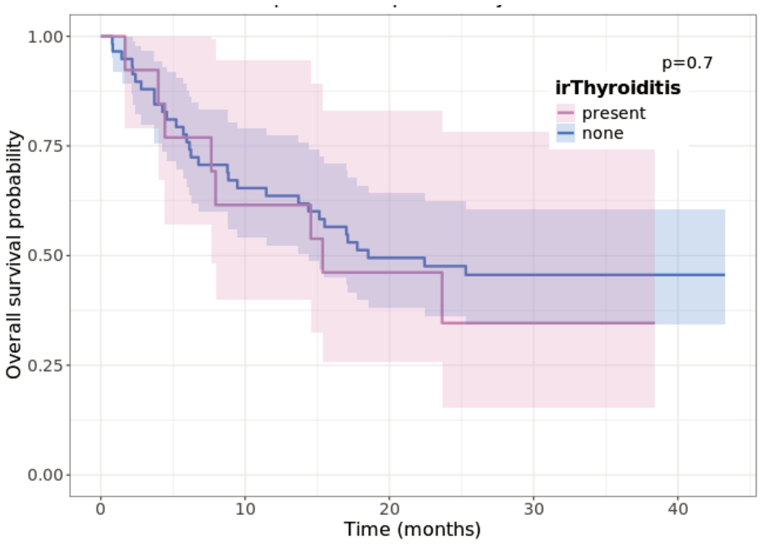

SUPPLEMENTARY FIGURE 1. Kaplan-Meier curves of median overall survival of metastatic melanoma patient, treated immune-checkpoint inhibitors by immune-related thyroiditis (irThyroiditis)
